# Genomic characterization of a Chinese bovine papillomavirus isolate and its L1-based phylogenetic placement in a global dataset

**DOI:** 10.3389/fvets.2026.1839050

**Published:** 2026-06-01

**Authors:** Yusheng Lin, Weiwei Liu, Jinxiu Jiang, Kul Raj Rai, Yongliang Che

**Affiliations:** 1 Institute of Animal Husbandry and Veterinary Medicine, Fujian Academy of Agricultural Sciences, Fuzhou, China; 2 NAST Biomedical Research Laboratory, Nepal Academy of Science and Technology (NAST), Lalitpur, Nepal

**Keywords:** bovine papillomavirus type 2, genomic characterization, molecular evolution, phylogenetic analysis, purifying selection

## Abstract

**Introduction:**

Bovine papillomatosis (BP), caused by bovine papillomavirus (BPV), is characterized by proliferative epithelial lesions and is associated with significant economic losses in cattle populations. Despite its clinical and economic relevance, the molecular characteristics of BPV isolates circulating in China, as well as their phylogenetic relationships within the context of global BPV genetic diversity, remain inadequately characterized.

**Methods:**

A BPV strain was isolated from a cattle farm in Fujian Province, China, and its complete genomic sequence was obtained. Using publicly available reference sequences, we analyzed the viral L1 gene through recombination screening, selection-pressure analysis (dN/dS ratio), and maximum-likelihood phylogenetic inference. Additional temporal signal analyses were conducted on the BPV2 subset to explore the presence of measurable temporal structure in the sequence data.

**Results:**

The newly identified strain, designated FJ-01, was classified as BPV2 and possessed a complete genome of 7,947 bp. No recombination was detected in the L1 gene. The overall dN/dS ratio indicated dominant purifying (negative) selection acting on the L1 gene (dN/dS = 0.0735). Maximum-likelihood phylogenetic analysis placed FJ-01 within the BPV2 clade in the final global L1 dataset. Exploratory temporal analyses of the BPV2 subset suggested weak temporal structure; however, estimates of absolute divergence dates were associated with high uncertainty and should be interpreted with caution.

**Discussion:**

This study provides a molecular characterization of a Chinese BPV isolate and integrates it into a global L1-based phylogenetic framework. Our findings support the genetic classification of FJ-01 within BPV2 and indicate the L1 gene is evolutionarily conserved. These results serve as a valuable reference for future molecular surveillance efforts and for more comprehensive studies utilizing geographically balanced and genome-complete BPV datasets.

## 1 Introduction

Papillomaviruses (PVs) belong to the family Papillomaviridae and are small, non-enveloped viruses characterized by a circular double-stranded DNA genome of approximately 8 kbp. The viral genome is organized into three primary functional regions; namely, a long control region (LCR) containing regulatory elements and two coding regions comprising early (E) and late (L) genes (1). PVs typically exhibit high host specificity and infect epithelial cells across a wide range of vertebrates (2).

Bovine papillomavirus (BPV) was first detected in 1929 and has since been reported in numerous countries, including Brazil, Mexico, and Iraq (3–6). BPV infection frequently causes benign papillomas on the skin, teats, and digestive tract. Although these lesions may regress spontaneously, they can also undergo malignant transformation in some cases, resulting in reduced livestock productivity and substantial economic losses, particularly in the leather and dairy sectors (7–9).

To date, 44 types of bovine papillomaviruses have been characterized and classified into five genera based on nucleotide sequence homology of the L1 gene (10). Among these, members of the genus Deltapapillomavirus including BPV1, BPV2, BPV13, and BPV14 are associated with fibropapillomas and exhibit a broader host range (11). BPV2, the genotype identified in this study, is particularly notable for its association with fibropapillomas that can undergo malignant transformation, especially in immunocompromised animals, underscoring the clinical importance of understanding its transmission dynamics.

In Fujian Province, the cattle population has exceeded 350,000 in recent years; nonetheless, the prevalence and genetic diversity of BPV remain poorly understood (12). Across China, molecular data on BPV are still limited, and the phylogenetic placement of Chinese isolates within global BPV diversity has not been sufficiently characterized (13, 14). Furthermore, although temporal and geographic analyses may provide useful evolutionary context, current inferences remain constrained by limited sampling density, uneven geographic representation, and the restricted temporal range of available sequences (15).

To address the identified knowledge gaps, this study analyzed a newly sequenced BPV strain from Fujian, China, alongside publicly available reference sequences. Specifically, we aimed to: (i) characterize the genomic features of the local isolate; (ii) determine its phylogenetic placement within a global L1 dataset; (iii) evaluate selective constraints acting on the L1 gene; and (iv) explore whether genotype-specific temporal and geographic patterns could be detected under the limitations of the currently available data.

## 2 Materials and methods

### 2.1 Virus identification and DNA extraction

In April 2023, a papilloma sample exhibiting a nipple-like morphology was collected from a one-year-old calf in Fujian Province, following informed consent obtained from the farmer. The tissue sample was homogenized and subjected to centrifugation. The resulting supernatant was then used for DNA extraction using the EasyPure® Simple Viral DNA/RNA Kit (TransGen, Beijing). Virus morphology was examined using transmission electron microscopy following negative staining, as previously described (16).

All animal procedures were conducted in accordance with the Regulations for the Administration of Affairs Concerning Experimental Animals, approved by the State Council of the People’s Republic of China. The study protocol was approved by the Research Ethics Committee of the Institute of Animal Husbandry and Veterinary Medicine, Fujian Academy of Agricultural Sciences (permit number 202307FJ-011).

### 2.2 Genotype identification and whole-genome amplification

Initial genotyping of BPV isolate was performed by polymerase chain reaction with universal primers MY11 and MY09, followed by Sanger sequencing and BLAST analysis using the GenBank database (17). Based on the reference BPV2 genome (GenBank accession no. MH187961.1), a set of primers was designed using Oligo 7 software to amplify the complete genome (Table 1). The resulting PCR products were purified, cloned into the pEASY®-Blunt Zero Vector (TransGen, Beijing), and subsequently transformed into competent *E. coli* DH5α cells. Bidirectional sequencing was performed on at least three clones per fragment, and the complete genome was assembled using DNASTAR 7.1.

**Table 1:** Primer sequences used for BPV whole-genome amplification.

Primer name	Primer sequence (5′ → 3′)	Product length
MY11	GGMCAGGGWCATAAYAATGG	455 bp
MY09	CGTCCMARRGGAWACTGATC	
BPV-FJ-1-F	ATGGACCTGCAAAGTTTTTC	3,575 bp
BPV-FJ-3560-R	CTATGGTTCTTTTTCACACG	
BPV-FJ-3440-F	AAGGCAGGAAGAAGAAGAAC	4,558 bp
BPV-FJ-7947-R	GTGGGAATCAGGGTCTGTCAGC	

### 2.3 Viral sequence dataset

Publicly available BPV reference sequences, along with their associated sampling information, were retrieved from the GenBank database and screened for eligibility in the comparative dataset (Table 2). Given that the L1 gene serves as the standard molecular marker for BPV classification and offers the broadest sequence comparability across independent studies, the L1 region was extracted from each qualifying genome or reference sequence. For subsequent phylogenetic reconstruction and selection pressure analyses, a final global L1 dataset comprising 90 sequences was compiled, which included the newly identified Chinese isolate. A subset of BPV2 sequences was additionally defined to facilitate exploratory temporal analyses. Multiple sequence alignment was performed using MAFFT version 7.520 (18), followed by manual refinement in MEGA version 11.0.13.

**Table 2:** Geographic and temporal distribution of 90 BPV sequences included in this study.

GenBank ID	Year	Country	Genotype
OP414244	2019	Belgium	BPV1
OP414186	2020	Belgium	BPV1
OP414223	2020	Belgium	BPV1
OP414224	2020	Belgium	BPV1
OP414225	2020	Belgium	BPV1
OP414232	2020	Belgium	BPV1
OP414236	2020	Belgium	BPV1
OP414247	2020	Belgium	BPV1
OP414248	2020	Belgium	BPV1
OP414261	2020	Belgium	BPV1
OP414270	2020	Belgium	BPV1
OP414181	2021	Belgium	BPV1
OP414188	2021	Belgium	BPV1
OP414190	2021	Belgium	BPV1
OP414227	2021	Belgium	BPV1
OP414234	2021	Belgium	BPV1
OP414246	2021	Belgium	BPV1
OP414269	2021	Belgium	BPV1
MF435918	2015	China	BPV1
KX907623	2016	China	BPV1
MF435917	2016	China	BPV1
MG263871	2017	China	BPV1
MK347523	2018	China	BPV1
OP414216	2020	France	BPV1
OP414263	2020	France	BPV1
OP414264	2020	France	BPV1
OP414192	2021	France	BPV1
OP414194	2021	France	BPV1
OP414203	2021	France	BPV1
OP414219	2021	France	BPV1
OP414237	2021	France	BPV1
OP414258	2021	France	BPV1
MG977494	2016	Italy	BPV1
MT119079	2017	Italy	BPV1
LC333380	2014	Japan	BPV1
LC510377	2017	Japan	BPV1
LC510378	2017	Japan	BPV1
LC510379	2017	Japan	BPV1
LC549663	2019	Japan	BPV1
KY746722	2016	Morocco	BPV1
PP855297	2023	Taiwan	BPV1
PQ374908	2024	Taiwan	BPV1
PQ374932	2024	Taiwan	BPV1
PQ381293	2024	Taiwan	BPV1
PQ381295	2024	Taiwan	BPV1
MH197482	2018	Turkey	BPV1
JX678969	2006	United Kingdom	BPV1
KU519390	2012	Brazil	BPV13
NC_030795	2012	Brazil	BPV13
MF741676	2014	Brazil	BPV13
KM258443	2014	China	BPV13
MG818475	2017	China	BPV13
MZ310895	2019	Belgium	BPV2
MZ310896	2019	Belgium	BPV2
MZ310897	2020	Belgium	BPV2
MZ310898	2020	Belgium	BPV2
MZ310899	2020	Belgium	BPV2
OP414221	2020	Belgium	BPV2
OP414233	2020	Belgium	BPV2
OP414235	2020	Belgium	BPV2
OP414243	2020	Belgium	BPV2
OP414250	2020	Belgium	BPV2
KU674833	2012	Brazil	BPV2
PQ140661	2021	Brazil	BPV2
KC878306	2012	China	BPV2
KM455051	2014	China	BPV2
OR130497	2023	China	BPV2
PP408177	2024	China	BPV2
OP414178	2020	France	BPV2
OP414230	2020	France	BPV2
OP414240	2020	France	BPV2
OP414185	2021	France	BPV2
OP414197	2021	France	BPV2
OP414199	2021	France	BPV2
OP414210	2021	France	BPV2
OP414211	2021	France	BPV2
OP414214	2021	France	BPV2
OP414215	2021	France	BPV2
OP414220	2021	France	BPV2
OP414228	2021	France	BPV2
OP414231	2021	France	BPV2
OP414251	2021	France	BPV2
OP414252	2021	France	BPV2
OP414255	2021	France	BPV2
OP414262	2021	France	BPV2
OP414268	2021	France	BPV2
LC510376	2017	Japan	BPV2
LC510383	2018	Japan	BPV2
LC510384	2018	Japan	BPV2
PQ381294	2024	Taiwan	BPV2

### 2.4 Saturation and recombination analyses

Substitution saturation within the L1 alignment was evaluated using DAMBE version 7 (19). Putative recombination events in the L1 gene were screened using seven distinct methods implemented in RDP version 4.100 (20); only events supported by at least four methods with a significance threshold of *p* < 10^−6^ were considered statistically significant. Additional validation was performed using SimPlot version 3.5.1 (21). As downstream evolutionary analyses were conducted exclusively on the L1 region, the absence of recombination inferred from this dataset was interpreted as specific to the L1 gene and was not generalized to the complete viral genome.

### 2.5 Selection pressure analysis

Selective pressure acting on the L1 gene in the final global dataset was evaluated using the SLAC, FEL, and FUBAR methods implemented on the Datamonkey server (22). To reduce false-positive rate, a codon site was considered to be under selection only when it was consistently supported by all three methods.

### 2.6 Phylogenetic analysis

Maximum-likelihood (ML) phylogeny of the final global L1 dataset was inferred using IQ-TREE version 2.3.1 (23). The best-fitting nucleotide substitution model was selected using ModelFinder (24). Branch support was assessed with 1,000 ultrafast bootstrap replicates and SH-aLRT support. The resulting ML tree served as the primary framework for genotype assignment and comparative phylogenetic interpretation.

### 2.7 Bayesian phylodynamic analysis

Temporal structure was first explored using TempEst version 1.5.3 (25). Because temporal signal was limited in the mixed-genotype dataset, subsequent Bayesian temporal analyses were restricted to the BPV2 subset. To further assess whether the sampling times contained non-random temporal information, a date-randomization test was performed on the BPV2 subset. Bayesian analyses were conducted in BEAST version 1.10.4 (26) under simplified tip-dating settings, employing a strict molecular clock and coalescent priors to explore genotype-specific temporal structure. Additional skyline-based analyses were examined as exploratory demographic analyses rather than as primary inferential models. Markov chain Monte Carlo (MCMC) chains were run for 5,000 generations, with samples taken at regular interval. Convergence and mixing were assessed using Tracer version 1.7.2, and effective sample sizes (ESS) were evaluated for parameter stability after discarding the first 10% of samples as burn-in. Maximum clade credibility (MCC) trees were summarized using TreeAnnotator version 1.10.4 and visualized in FigTree version 1.4.4 where appropriate. Because deep-node date estimates remained unstable, Bayesian temporal results were interpreted cautiously and were used primarily to assess temporal signal rather than to support precise absolute dating.

### 2.8 Phylogeographic analysis

Exploratory phylogeographic reconstruction was performed for the BPV2 subset in BEAST using a discrete diffusion model with asymmetric transition rates (27). Bayesian stochastic search variable selection (BSSVS) was used to identify supported migration pathways. Because geographic sampling was uneven across regions, phylogeographic results were interpreted cautiously and emphasis was placed on only the strongest supported transitions, whereas lower-support pathways were treated as tentative. Results were visualized in SpreaD3 version 0.9.7.1 (28).

### 2.9 Compliance with ARRIVE guidelines

This study complied with the ARRIVE guidelines (Animal Research: Reporting of *In Vivo* Experiments) for animal studies. The 3R principles (Replacement, Reduction, Refinement) were strictly followed throughout the experimental procedures. Specifically, tissue samples were collected from naturally occurring lesions during routine farm management, thereby avoiding the need for experimental induction of tumors in healthy animals (Replacement). The number of animals sampled was reduced to the minimum required for successful virus isolation and genomic characterization, with only one calf providing the sample for this study (Reduction). All sampling procedures were performed by experienced veterinarians using aseptic techniques and appropriate local anesthesia to minimize pain and distress, and animals were closely monitored post-sampling until full recovery (Refinement).

## 3 Results

### 3.1 Virus morphology and genome characterization

**Figure 1:**
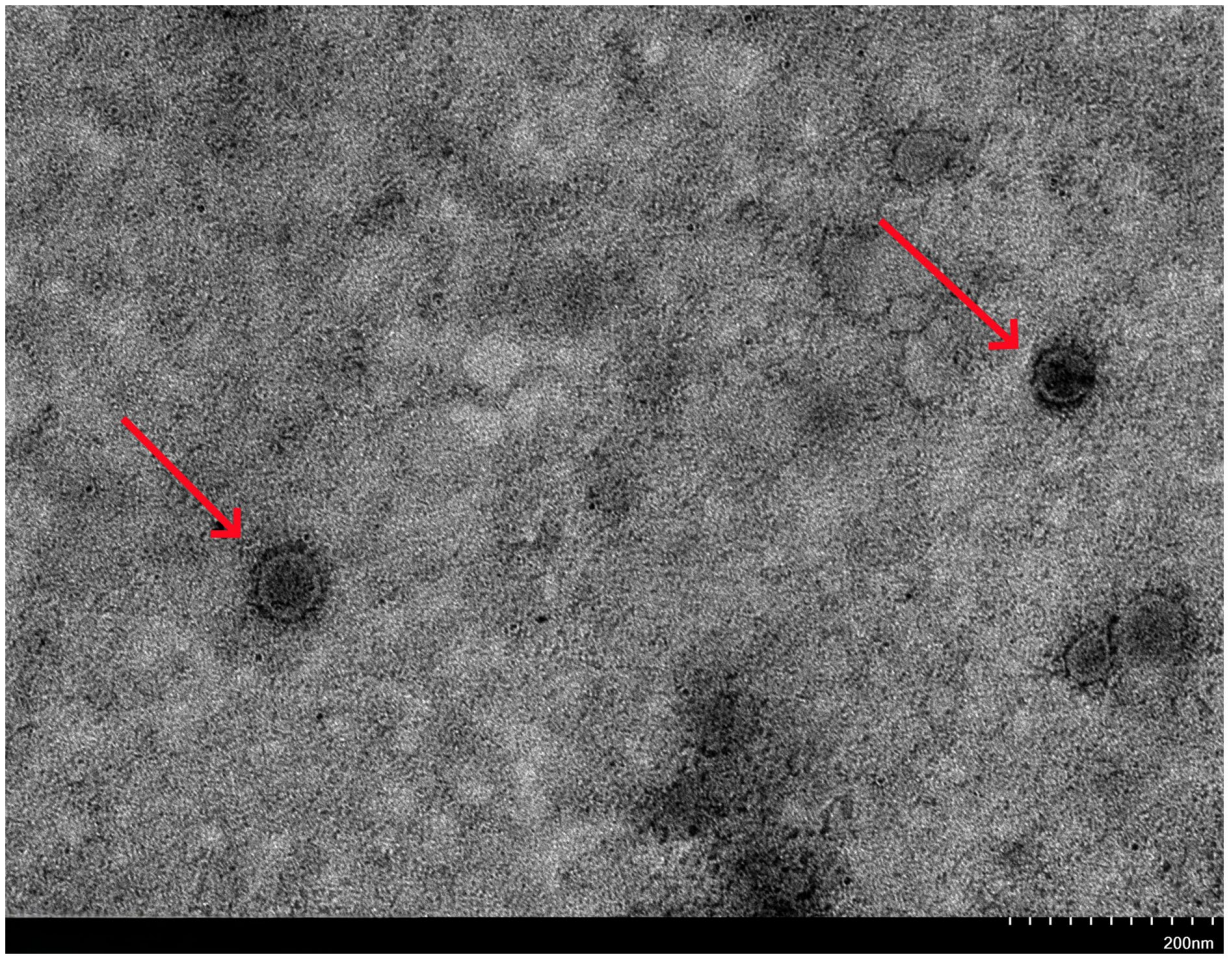
Transmission electron micrograph of BPV particles after negative staining. Virus particles are indicated by red arrows.

Transmission electron microscopy revealed spherical virus particles approximately 55 nm in diameter (Figure 1). The complete genome of strain FJ-01 (GenBank accession no. OR130497) was 7,947 bp in length with a GC content of 45.93%. It contained nine open reading frames (ORFs): E1, E2, E4, E5, E6, E7, E8, L2, and L1. BLAST analysis showed the highest nucleotide identity (99.7%) to the Brazilian strain BR/02 AC12 (GenBank: MH187961).

### 3.2 Sequence saturation and recombination

Saturation analysis yielded an ISS value of 0.0994 (ISS.c = 0.7679, *p* = 0), indicating that the L1 alignment had not reached substitution saturation. No recombination events were detected in the L1 gene by either RDP or SimPlot. These results supported the use of the final L1 dataset in downstream phylogenetic and selection-pressure analyses.

### 3.3 Selection pressure analysis

Selection pressure analysis showed that the L1 gene was predominantly subject to purifying selection. SLAC estimated an overall dN/dS ratio of 0.0735. At the selected thresholds, SLAC detected 24 negatively selected sites and no positively selected sites, FEL identified 1 positively selected site and 175 negatively selected sites, and FUBAR identified 3 positively selected sites and 215 negatively selected sites. However, no positively selected site was consistently supported by all three methods (Table 3).

**Table 3:** Selection pressure analysis of BPV L1 protein.

Model	L1 protein		
	SLAC	FEL	FUBAR
dN/dS	0.0735		
Numbers of positive selection site	0	1	3
Numbers of purifying selection site	24	175	215

### 3.4 Phylogenetic analysis

**Figure 2:**
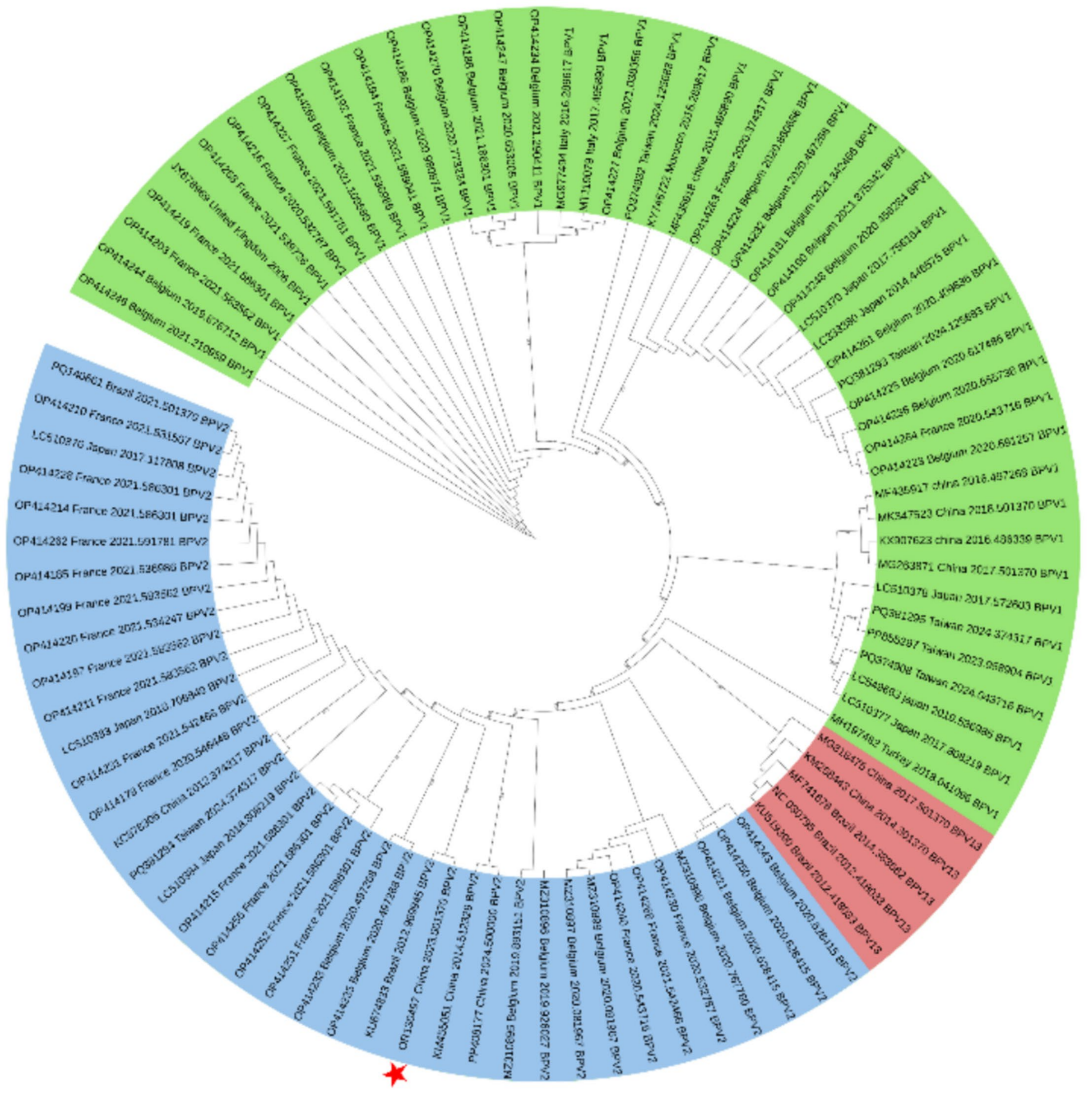
Maximum-likelihood phylogenetic tree of 90 BPV sequences based on the L1 gene. Tip labels include GenBank accession, country of origin, and genotype. Different colors represent distinct genotypes: blue (BPV1), red (BPV2), and purple (BPV13). Node support values are shown as SH-aLRT/ultrafast bootstrap where appropriate. The newly isolated FJ-01 strain is indicated in red.

Maximum-likelihood phylogenetic analysis of the final global L1 dataset grouped the sequences into three major genotype-defined clusters: BPV1, BPV2, and BPV13, with generally strong branch support (Figure 2). The newly identified strain FJ-01 clustered within the BPV2 clade, confirming its genotype assignment and phylogenetic placement within the global L1 framework.

### 3.5 Temporal dynamics and population history

Root-to-tip regression of the mixed-genotype dataset indicated only limited temporal structure (R^2^ = 0.1073; correlation = 0.3276), suggesting that absolute time calibration should be interpreted cautiously. We, therefore, further evaluated temporal signal within the BPV2 subset. Date-randomization testing under a strict-clock, constant-size coalescent model showed clear separation between the clock-rate estimate from the observed sampling dates and those from 10 randomized datasets, supporting the presence of temporal signal. However, the posterior uncertainty of the real dataset remained broad, indicating that the temporal signal was detectable but moderate. Under the same framework, the mean substitution rate was estimated at 7.724 × 10^−5^substitutions/site/year. Because deep-node age estimates remained unstable, Bayesian temporal analyses were treated primarily as supportive assessments of genotype-specific temporal structure rather than as a basis for precise tMRCA inference. Exploratory skyline analyses were also performed, but because key skyline parameters showed limited convergence, these results were interpreted cautiously and were not used as the main basis for demographic conclusions.

### 3.6 Phylogeographic patterns

Phylogeographic reconstruction of the BPV2 dataset indicated a complex diffusion history rather than a simple unidirectional spread from a single region. In the MCC summary tree, the root state was reconstructed as Brazil, but with only moderate support (location.prob. = 0.5315). Several deeper internal nodes were reconstructed as China, also with only moderate support (location.prob. ≈ 0.71–0.77), indicating substantial uncertainty in ancestral geographic states at deeper nodes. By contrast, France and Belgium formed the major sampled backbone of BPV2 diversity, whereas Chinese sequences were distributed across multiple positions in the tree rather than forming a single compact monophyletic cluster. BSSVS analysis identified only a limited number of well-supported migration links. The strongest supported route was Belgium → France (Bayes factors [BF] = 133.46), followed by Brazil → China (BF = 26.23), France → Brazil (BF = 24.19), China → Belgium (BF = 14.54), Japan → France (BF = 13.30), and China → Japan (BF = 11.26) (Table 4). Most other links showed low support and were therefore not interpreted further. Overall, these results suggest that Europe, particularly France and Belgium, functioned as an important dispersal hub in the sampled BPV2 dataset, but these results do not support assigning Europe as the definitive ancestral source.

**Table 4:** Well-supported migration pathways identified by BSSVS phylogeographic analysis of the BPV2 subset.

From	To	Bayes factor	Posterior probability	Mean rate
Belgium	France	133.46	0.969	1.187
Brazil	China	26.23	0.860	1.256
France	Brazil	24.19	0.850	1.198
China	Belgium	14.54	0.773	1.163
Japan	France	13.30	0.757	1.199
China	Japan	11.26	0.725	0.913

## 4 Discussion

This study integrates a newly sequenced BPV strain from China with a global comparative dataset to characterize its genomic features and phylogenetic placement within BPV diversity. Rather than offering definitive evidence for global transmission drivers, our L1-based evolutionary framework allows for cautious exploration of temporal and phylogeographic patterns within the constraints of the currently available data.

The L1 gene appears to evolve under strong purifying selection, consistent with its important structural role in viral capsid assembly and host cell entry (29). The low dN/dS ratio observed in the final dataset supports the view that L1 is evolutionarily conserved and remains an informative marker for BPV classification and comparative phylogenetic analysis (29, 30).

Although exploratory temporal analyses of the BPV2 subset suggested the presence of some temporal structure, absolute age estimates remained unstable and should not be interpreted as precise historical dates. The currently available sampling span is limited, and additional historical, archival, or more densely sampled contemporary sequences will be required before deep divergence times can be estimated with confidence. Although the BPV2 subset retained detectable temporal information, the signal was still relatively weak, and absolute estimates such as tMRCA should be regarded as approximate rather than precise.

The phylogeographic results should be interpreted cautiously. Although France and Belgium accounted for a large proportion of the sampled BPV2 diversity and formed the main backbone of the MCC tree, the ancestral geographic history was not strongly resolved. The root state in the MCC tree was inferred as Brazil with only moderate support, and several deeper ancestral nodes were reconstructed as China, also with only moderate support. Taken together, these findings indicate that BPV2 spread was geographically complex and cannot be reduced to a simple Europe-to-Asia scenario (31). Therefore, Europe is better interpreted as an important inferred hub within the available dataset rather than an unequivocal origin of BPV2. Likewise, any association between the inferred migration links and historical livestock movement should be regarded as plausible but indirect, because trade variables were not formally incorporated into the phylogeographic model.

### 4.1 Clinical relevance of BPV2 transmission

BPV2, the genotype identified in our newly sequenced strain FJ-01, is clinically important because of its association with fibropapillomas and its potential for malignant progression, including urinary bladder tumors, in susceptible animals (9, 11). The phylogenetic placement of FJ-01 within the BPV2 clade confirms that clinically relevant BPV2 lineages are present in Chinese cattle. This finding supports continued surveillance in herds with persistent papillomatosis or other risk factors for severe disease, particularly in regions where BPV2 circulation may be underrecognized.

### 4.2 Implications for vaccine and diagnostic development

The strong purifying selection observed in the L1 gene has important implications for vaccine and diagnostic development. Because L1 forms the major capsid protein and remains comparatively conserved, it continues to represent a useful target for molecular detection and for evaluating antigenic stability across BPV lineages (32, 33). From a diagnostic perspective, the conservation of L1 supports the use of L1-targeted assays for broad BPV detection, whereas the phylogenetic clustering observed in this study suggests that genotype-specific assays or confirmatory sequencing may be required to distinguish among BPV1, BPV2, and BPV13 in clinical settings (30).

### 4.3 Potential surveillance and control implications

Although our analyses do not formally demonstrate the role of livestock trade, they suggest that animal movement may contribute to the geographic distribution of BPV lineages, with practical implications for surveillance and control. First, enhanced surveillance of imported cattle through pre-movement screening, quarantine, and molecular testing may reduce BPV introduction risk in high-movement settings. Second, integrating sequence-based surveillance into routine diagnostics may enable early detection of lineage turnover, persistence, or introductions, with phylogenetics complementing conventional epidemiology. Third, the observed genotype composition (BPV1, BPV2, and BPV13) supports continued attention to BPV1 and BPV2 for vaccine-oriented surveillance, while BPV13 also warrants monitoring; more balanced sequence data will allow reassessment of genotype composition to inform vaccine design. Fourth, biosecurity measures, including transport hygiene, disinfection of shared equipment, and temporary segregation of new animals, may reduce inter-herd transmission opportunities.

### 4.4 Broader implications

Although BPV is not zoonotic, studies of its genetic diversity and lineage distribution may still offer a useful comparative framework for understanding the movement of livestock-associated pathogens. More broadly, integrating molecular surveillance with animal movement and herd-management data may strengthen risk-based monitoring of transboundary animal diseases.

### 4.5 Comparison with human papillomavirus evolution

The evolutionary behavior of BPV shares several broad features with human papillomavirus (HPV), including slow sequence evolution and strong purifying selection. However, because the present study is based on the BPV L1 gene and limited temporal structure was detected outside the BPV2 subset, direct comparisons of deep evolutionary timescales should be made cautiously (29, 32).

### 4.6 Limitations and future directions

Several limitations should be acknowledged. First, the dataset remained uneven across genotypes and geographic regions, with stronger temporal support for BPV2 than for the mixed-genotype dataset as a whole. Second, the use of the L1 gene alone does not exclude recombination or distinct evolutionary processes in other genomic regions (34, 35). Third, incomplete geographic sampling may affect the stability of inferred migration pathways. Fourth, the available sampling span was insufficient to support robust deep-time calibration. Future studies should incorporate more geographically balanced datasets, additional complete genomes, and denser temporal sampling to improve inference of BPV evolution and dispersal. Functional studies comparing the biological properties of major BPV genotypes would also complement the sequence-based results presented here.

## 5 Conclusion

This study characterizes a newly identified Chinese BPV isolate, FJ-01, within a global L1-based phylogenetic framework. The isolate was assigned to BPV2, and the L1 gene was found to be evolutionarily conserved under strong purifying selection. Although exploratory analyses suggested detectable temporal and phylogeographic structure within BPV2, these inferences remain constrained by sampling limitations and dating uncertainty. These findings provide a molecular reference for BPV surveillance in China and highlight the importance of larger, more balanced, and genome-complete datasets for future phylodynamic studies.

## Data Availability

The complete genome sequence of strain FJ-01 has been deposited in GenBank under accession number OR130497. All alignment files, BEAST XML input files, and phylogenetic trees generated during this study are available from the corresponding author upon reasonable request. The datasets analyzed in this study are publicly available in the GenBank repository with accession numbers listed in Table 2.
